# A comprehensive evaluation between dexmedetomidine and midazolam for intraoperative sedation in the elderly: protocol for a systematic review and meta-analysis of randomized controlled trials

**DOI:** 10.1186/s13643-022-02144-7

**Published:** 2022-12-23

**Authors:** Chunxia Huang, Zunjiang Li, Yingxin Long, Dongli Li, Manhua Huang, Banghan Ding, Wei Zhu

**Affiliations:** 1 The Second Clinical College, Guangzhou University of Chinese Medicine, Guangzhou, China; 2 The Second Affiliated Hospital of Guangzhou University of Chinese Medicine, Guangzhou, 510120 China

**Keywords:** Dexmedetomidine, Midazolam, Meta-analysis, Intraoperative sedation, Randomized controlled trials

## Abstract

**Background:**

The sedative effect of intraoperative sedation in elderly surgery exerts critical influence on the prognosis. Comparison on the safety and efficacy between dexmedetomidine and midazolam in many clinical randomized controlled trials (RCTs) was inconsistent and suspicious. We aim to comprehensively evaluate the safety and efficacy between dexmedetomidine and midazolam for intraoperative sedation in the elderly via meta-analysis and systematic reviews.

**Methods:**

RCTs regarding to the comparison of sedative effects and safety between dexmedetomidine and midazolam in elderly patients (aged ≥ 60 years) will be comprehensively searched from 2000 October to 2022 May through 4 English databases and 4 Chinese databases. After extraction in duplicate, the systematic review and meta-analysis will be performed on the primary outcomes (hemodynamic changes, sedative effect, cognitive function) and secondary outcomes (analgesic effect, surgical characteristics, complications, or adverse reactions) for assessing the two therapy methods using Review Manager software (Version 5.3). Sensitivity analysis will be conducted to evaluate the heterogeneity of the results; funnel plot and Egger’s trial will be performed to analyze publication bias of the included studies, and trial sequential analysis will be applied to assess the robustness and reliability of preliminary meta-analysis results. Finally, rating quality of evidence and strength of recommendations on the meta results will be summarized by Grading of Recommendations, Assessment, Development and Evaluations (GRADE) approach.

**Discussion:**

This systematic review and meta-analysis will evaluate the safety and efficacy between dexmedetomidine and midazolam for intraoperative sedation in the elderly; it will give an insight on the application of dexmedetomidine and midazolam and will provide evidence-based reference for clinical decision-making.

**Systematic review registration:**

PROSPERO CRD42021221897

## Background

In clinical practice, elderly population (aged ≥ 60 years) suffers from increased risk of diseases due to their reduced physical function and weakened immune function, and they are increasingly dependent on surgical treatment [1, 2]. Older people have surgery at a higher rate than other age groups, making up 15% of the total population [3]. Despite significant advances in surgery and anesthesia, anesthetics can still affect the outcome of disease treatment. Therefore, the better and more rational anesthetic medication in elderly surgery attracts increasing concern and interest.

Intraoperative sedation, often used to improve patient comfort, also regarded as surgical anesthesia [4, 5]. In order to reduce the occurrence of postoperative complications, safe and effective anesthetics are issues that need be considered in clinic. Dexmedetomidine is a highly selective α2-adrenergic receptor agonist; its sedation and analgesic effects are similar to opioids [6–9]. Dexmedetomidine provides a unique “conscious sedation” in which the patient appears to be sleeping and easily aroused, and the sedation and breathing patterns it provides are consistent with natural sleep [10, 11]. Dexmedetomidine is an important option for sedation in intensive care [12]. Dexmedetomidine applied in anesthesia can prolong the duration of sensory and motor block and reduce the cumulative analgesic dosage for 24 h after surgery [13]. Midazolam, belongs to midazolam benzodiazepines, represses the excitatory response combined with γ-aminobutyric acid receptors [14]. It is widely used for hypnotic, anticonvulsant, sedation, anxiolytic, and amnesia [15]. Midazolam has a high affinity for benzodiazepine receptors in the central nervous system, with data showing twice the affinity of diazepam [16]. Midazolam prevents autonomic, hormonal, and circulatory adverse effects without causing nausea and vomiting [17, 18]. Thus, midazolam and dexmedetomidine were the subjects of study among numerous anesthetics. However, studies have shown that sudden cessation of dexmedetomidine can lead to clonidin-like withdrawal syndromes, including tension, headache, and agitation [19]. The specific antagonist atipamezole can easily reverse the situation [20]. It is reported that dexmedetomidine application may result in intraoperative comorbidities including uncontrollable hypotension, severe bradycardia, and asystole [21, 22]. Studies have also shown that midazolam exerts fast anesthetic effect within 5 min after intramuscular injection, but it may cause loss of airway reflex, respiratory depression and even apnea during anesthesia, etc. [23].

The elderly population is at risk of serious complications during the operation and needs special care [24]. It is necessary to compare the safety of effective sedatives for the elderly. No reliable and consistent conclusions have been reached regarding dexmedetomidine and midazolam for sedation in elderly patients. Only a few meta-analyses related to anesthesia in children have confirmed that dexmedetomidine is safer and more effective than midazolam in sedation and reducing postoperative complications [25–27]. However, there still lacks comparison on the safety and efficacy between dexmedetomidine and midazolam in elderly surgery due to significant heterogeneities between the clinical RCTs, resulting in inconsistent and controversial conclusions. Some studies also reported that there is no significant difference in the sedation between dexmedetomidine and midazolam [28–30]; thus, the conclusions of these researches have been questioned to a certain extent.

This study is designed to compare the safety and sedative effects between dexmedetomidine and midazolam in elderly patients with surgery. In order to minimize the heterogeneity and bias, we will select RCTs addressing the safety and sedative effects of dexmedetomidine and midazolam in elderly patients. Systematic reviews and meta-analysis represent an appropriate design to elucidate the clinical usage and efficacy of dexmedetomidine and midazolam in previous RCTs, providing clinicians and policymakers with an overall assessment of the evidence on the safety and sedative effects between dexmedetomidine and midazolam, which is necessary for both practitioners and elderly patients.

## Methods

### Study design and objectives

The program will be designed according to the Preferred Reporting Items for Systematic Review and Meta-Analysis Protocol (PRISMA-P) [31]. It has been registered in the PROSPERO database (CRD42021221897). It aims to provide a systematic review on the effect between dexmedetomidine and midazolam in elderly patients. Additionally, meta-analysis of RCTs will be performed in order to assess the safety and efficacy of dexmedetomidine and midazolam on primary and secondary outcomes of RCTs.

### Eligibility criteria

#### Types of participants

Patients with surgery age more than 60 or older and receive anesthetics intervention will be included, whose ASA status is I-III. Regardless of sex, region, race, and the type of surgery, as detailed in Table 1, the MESH term of population will include “The elderly,” “The old man,” “The old patient,” “The elderly patients,” “The aged” and “Intraoperative sedation,” “Sedation,” “Surgery sedation,” “Surgical sedation,” “Anesthesia and analgesia,” “Anesthesia sedation,” “Intraoperative anesthesia,” “Surgery anesthesia,” and “Surgical anesthesia.”

**Table 1:** Patients, Interventions, Comparisons, Outcomes, Study design (PICOS) framework

PICOS	Searched items
Patients (a + b)	
a	The elderly; The old man; The old patient; The elderly patients; The aged
b	Intraoperative sedation; Sedation; Surgery sedation, Surgical sedation; Anesthesia and analgesia; Anesthesia sedation; Intraoperative anesthesia; Surgery anesthesia; Surgical anesthesia
Interventions	Dexmedetomidine, Dexmedetomidine Hydrochloride, Hydrochloride Dexmedetomidine
Comparisons	Midazolam, Benzodiazepine, Compounds, Benzodiazepine, Benzodiazepinones, devazepide
Outcomes	
Primary outcomes	Hemodynamic changes (MAP, HR, SBP, DBP, RR, SPO2), Sedative effect (OAA/S, BIS), and Cognitive function (MMSE, POCD)
Secondary outcomes	Analgesic effect (RASS, VAS), Surgical characteristics (operation time, the volume of intraoperative blood loss, time of extubation), Complications or adverse reactions (agitation, hypotension, bradycardia, respiratory depression, postoperative nausea and vomiting, bradycardia and shivering)
Studies design	Random control study, Control study, Random control trial, Clinical control trial, Clinical trial, Randomized control trial, Randomized controlled trial

#### Types of intervention in the experimental group and control group

Patients in the experimental group and control group received dexmedetomidine or midazolam, respectively, with or without the same conventional treatment other anesthetics. The MESH term of interventions included “Dexmedetomidine,” “Dexmedetomidine Hydrochloride” and “Hydrochloride Dexmedetomidine” in the experimental group, and “Midazolam,” “Benzodiazepine,” “Benzodiazepine,” and “Benzodiazepinones, devazepide” in the control group (Table 1).

#### Type of included indicators or outcomes

The primary results will contain hemodynamic changes (mean arterial pressure (MAP), heart rate (HR), systolic blood pressure (SBP), diastolic blood pressure (DBP), saturation of pulse oxygen (SPO2)), sedative effect (observer assessment of alertness/sedation scale (OAA/S), bispectral index (BIS), Richmond Agitation-Sedation Scale (RASS)) and cognitive function (mini-mental state examination (MMSE), postoperative cognitive dysfunction (POCD)). The secondary results will include analgesic effect (Visual Analogue Scale/Score (VAS)), surgical characteristics (operation time, the volume of intraoperative blood loss, and extubation time), complications, or adverse reactions (hypotension, agitation, postoperative nausea and vomiting, bradycardia, respiratory depression, delirium, and shivering).

### Search strategy

Four English databases (PubMed, Embase, Web of Science, Cochrane Library) and four Chinese databases (Chinese Biomedical Literature Database, Chinese Science and Technology Journals VIP Database, China National Knowledge Infrastructure (CNKI), Wan-fang Database) will be searched for literatures published between October 2001 and May 2022. The search strategy will be conducted according to the patients, interventions, comparisons, outcomes, and study design (PICOS) components (Table 1). The complete search strategy will use a combination of PICOS framework (P+I+C+O+S or P+I+C). Only clinical randomized controlled trials (RCTs) published in English or Chinese will be included. Search strategy in PubMed is shown in Table 2. Any differences can be resolved through discussion with other reviewers (ZW, DBH).

**Table 2:** Search strategy of PubMed for randomized control trials

Searched strategy in PubMed	
Block 1: The elderly	
#1	“The elderly” [tiab]
#2	“The old man” [tiab]
#3	“The old patient” [tiab] OR “The elderly patients” [tiab]
#4	“The aged” [tiab] OR “The greybeard” [tiab]
#5	#1 OR #2 OR #3 OR #4
Block 2: Intraoperative sedation	
#6	“Intraoperative sedation” [Mesh] OR “Intraoperative anesthesia” [Mesh] OR “Intraoperative analgesia” [Mesh]
#7	“Surgical sedation” [tiab]
#8	“Surgical anesthesia” [tiab] OR “Surgical analgesia” [tiab]
#9	#6 OR #7 OR #8
Block 3: Participants	
#10	#5 AND #9
Block 4: Intervention in experimental group	
#11	“Dexmedetomidine” [Mesh]
#12	“Dexmedetomidine hydrochloride” [tiab] OR “Hydrochloride, dexmedetomidine” [tiab] OR “Dexmedetomidine” [tiab]
#13	#11 OR #12
Block 5: Intervention in control group	
#14	“Midazolam” [Mesh]
#15	“Benzodiazepine” [tiab] OR “Benzodiazepines” [tiab]
#16	“Azepines” [tiab]
#17	“Hydrochloride, midazolam” [tiab] OR “Midazolam hydrochloride” [tiab]
#18	“Midazolam maleate” [tiab] OR “Maleate, midazolam”
#19	#14 OR #15 OR #16 OR #17 OR #18
Block 6: Study type	
#20	“Randomized controlled trial” [pt]
#21	“Controlled clinical trial” [pt]
#22	“Randomized” [tiab] OR “randomly” [tiab] OR “trial” [tiab] OR “groups” [tiab]
#23	#20 OR #21 OR #22
Block 6: Final merge	
#24	#10 AND #13 AND #19 AND #23

### Study selection

**Fig. 1:**
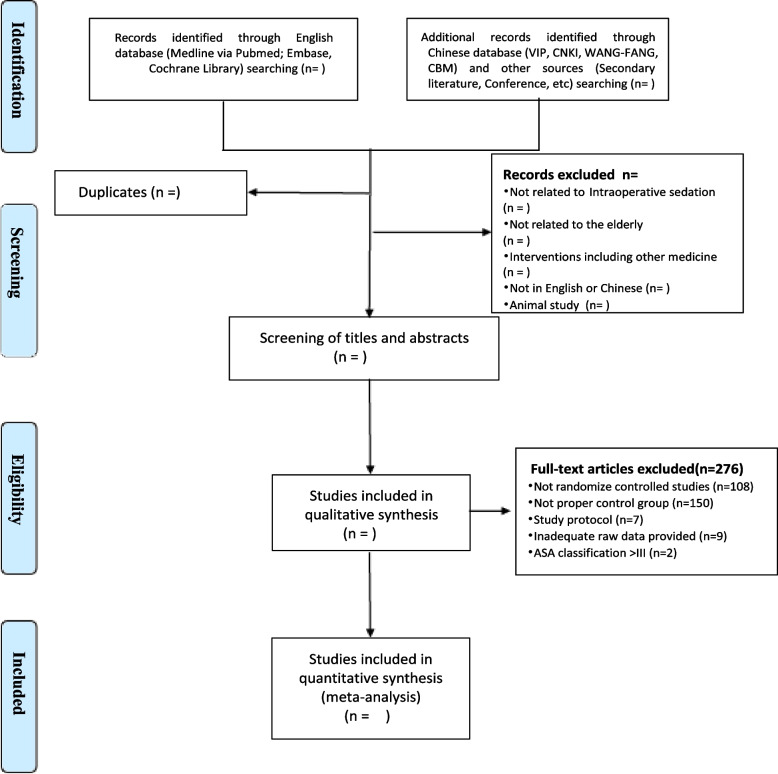
PRISMA flow chart depicting studies included in the systematic review and meta-analysis

Two independent reviewers (HCX, LZJ) will screen relevant trials by examining titles and abstracts of the identified studies. Any studies that do not conform to the PICOS framework will be excluded. Full articles will be obtained and checked for extracted details (LYX, LDL). Any reason for extraction will be recorded. Possible divergency will be resolved by discussion or a third reviewer (DBH). The selection process will be presented in a PRISMA flow diagram (Fig. 1).

### Data extraction and exclusion criteria

We will extract each qualified data. Data will be extracted including year of publication, author’s name, study type, randomized methodology, and basic characteristics of participants (ASA classification, mean age, sample size, type of surgery, body mass index/weight, duration of anesthesia, duration of surgery, medication use, and outcomes) (Tables 3 and 4).

**Table 3:** Characteristics of included RCTs assessing effect of dexmedetomidine and midazolam for elderly surgery patients (e.g.)

No.	Author(year)	Type of study (random method)	ASA status	Sample size (M/F)		Age (year)		BMI/weight(kg)	
				D	M	D	M	D	M
1	Wenfei Tan (2016)	Double-blind RCT (sealed envelopes)	I-III	22	22	70.1 ± 7.0	73.1 ± 9.3	23.5 ± 3.9	22.9 ± 2.3
2									
3									

Note: *ND* not described in the study, *D* dexmedetomidine, *M* midazolam, *ASA* status American Society of Anesthesiologists physical status, *M/F* male/female, *BMI* body mass index

**Table 4:** Characteristics of included RCTs assessing effect of dexmedetomidine and midazolam for elderly surgery patients (e.g.)

No.	Author (year)	Anesthesia duration		Operation duration		Dosage and usage of D		Dosage and usage of M		Type of disease
		D (min)	M (min)	D (min)	M (min)	Anesthesia induction	Anesthesia maintenance	Anesthesia induction	Anesthesia maintenance	
1	Wenfei Tan (2016)	91.9 ± 28.0	99.9 ± 46.3	69.9 ± 23.4	78.2 ± 46.9	1 μg/kg for 10 min	12 μg/(kg·h)	22.5 μg/(kg·h) for 10 min	45 μg/(kg·h)	Elective transurethral prostatic resection
2										
3										

Note: *ND* not described in the study, *NOT* no dosage and usage of anesthesia, *D* dexmedetomidine, *M* midazolam

Nonhuman trials, non-peer-reviewed publications, retrospective observational studies, prospective observational studies, review article, case reports, cohort studies, and letters to the editor will be excluded. Any other factors that do not meet with the inclusion criteria will also be excluded as well.

### Risk-of-bias assessment

The updated ROB2 tool according to the instructions will be used for risk-of-bias assessment (https://methods.cochrane.org/bias/resources/rob-2-revised-cochrane-risk-bias-tool-randomized-trials). The criteria for risk of bias will be assessed according to randomization process, deviations from the intended interventions, missing outcome data, measurement of the outcome, and selection of the reported result. Quality assessments will be performed by two independent authors, and the results will be rated as “high risk,” “some concerns,” or “unclear” risk of bias. Discussions will be held by all authors to resolve any disagreements.

### Statistical analysis

The effect size will be pooled using the Review Manager software tool (RevMan, v.5.3; The Cochrane Collaboration). Fixed-effect model will be used for the pool with low heterogeneity, and random-effects model will be used for the pool with high heterogeneity. Mean deviation (MD) or standard mean difference (SMD) and 95% confidence intervals (CI) will be utilized for continuous data, and relative risk (RR) with 95% CI will be calculated for dichotomous data. Subgroup analysis and sensitivity analysis will be also used to investigate potential sources of heterogeneity.

### Sensitivity analysis and subgroup analysis

When it occurs relatively high heterogeneity of the results, sensitivity analysis will be performed to explore the source of high heterogeneity in order to enhance the credibility of the results. Any possibility (methodological quality, heterogeneity, studies quality, surgery duration, number of samples etc.) resulted in high heterogeneity will be considered for subgroup analysis.

### Potential publication and reporting bias analysis

After preliminary meta-analysis, potential publication bias will be assessed visually using funnel plots, and Egger’s regression test and Begg’s test will be utilized to detect the funnel plot asymmetry.

#### Trial sequential analysis (TSA)

For further illustrating and confirming the reliability of final results, trial sequential analysis will be performed on the analyzed outcomes via TSA software (version 0.9.5.10 Beta; Copenhagen Trial Unit, Center for Clinical Intervention Research, Rigshospitalet, Copenhagen, Denmark), aiming to rule out the possibility of false positives and confirm firm evidence of final results.

### Rating quality of evidence and strength of recommendations

Finally, we will conduct evaluation on the quality of evidence regarding to the main positive outcomes using the GRADEprofiler 3.6.1 evaluation tool. Briefly, the quality of the evidence will be divided into four levels: high, medium, low, and very low, and strength of recommendations will be divided into strong recommendation and weak recommendation. RCT trials will be regarded as high quality of evidence, and then, the evidence quality level of the outcomes will be assessed from the five downgrading factors of research limitations, inconsistency, indirectness, imprecision, and publication bias.

## Discussion

Secure and effective anesthetics effectively reduce the occurrence of postoperative complications and improve perioperative cognitive dysfunction or intraoperative awareness, providing clinicians with better surgical procedures [32]. However, appropriate anesthetic for elderly patients with surgery is still a serious challenge for clinicians [33].

Up to date, there is not enough evidence to determine a better anesthesia plan for elderly patients [34].

Various drugs including ketamine, propofol, benzodiazepines, fentanyl, etomidate, and dexmedetomidine exert intraoperative sedation. Midazolam and dexmedetomidine is both applied for anesthesia and sedation in the elderly [35]. However, studies have indicated that midazolam led to cognitive impairment and postoperative or delirium in the elderly [36]. Although dexmedetomidine does not directly damage the respiratory system and cause apnea, it has been shown to cause hypoxia and hypercapnia [36]; it can also lead to hemodynamic effects such as hypertension, hypotension, and bradycardia, whereas, in some clinical studies, their conclusions are inconsistent and suspicious due to their heterogeneity in research methods [37, 38]. Up to date, it still lacks comprehensive acknowledgement on the safety and efficacy of dexmedetomidine and midazolam.

In conclusion, systematic evaluation on the efficacy and safety of dexmedetomidine and midazolam for intraoperative sedation in elderly patients can provide the anesthesiologist with insight to the appropriate anesthetic choice, aiming to reducing the potential postoperative complications of elderly patients [34]. This protocol maximizes the extraction of relevant information with a clear and structured procedure; however, we also acknowledge several limitations existing in this systematic review and meta-analysis. The assessment methods differ in different included studies may result in medium or high heterogeneity. Some of the included studies may be of low quality. Moreover, only studies published in English and Chinese will be included. Therefore, we will continue to pay attention to update our conclusions in future.

## Data Availability

The datasets used and/or analyzed during the current study are available from the corresponding author on reasonable request.

## Abbreviations

RCTs: Randomized controlled trials
RASS: Richmond Agitation-Sedation Scale
MMSE: Mini-mental state examination
PICOS: Patients, interventions, controls, outcomes, and study design
BIS: Bispectral index
VAS: Visual Analogue Scale/Score
HR: Heart rate
ASA: American Society of Anesthesiologists
SMD: Standard mean difference
MAP: Mean arterial pressure
SBP: Systolic blood pressure
DBP: Diastolic blood pressure
OAA/S: Observer assessment of alertness/sedation scale
POCD: Postoperative cognitive dysfunction
SPO2: Saturation of pulse oxygen
OP: Odds ratio
95% CI: 95% confidence intervals
TSA: Trial sequential analysis
